# Fatty acid malabsorption followed by chylomicron malformation, not pancreatic insufficiency, cause metabolic defects in cystic fibrosis

**DOI:** 10.1016/j.jlr.2024.100549

**Published:** 2024-04-21

**Authors:** Gary F. Lewis

**Affiliations:** Division of Endocrinology, Departments of Medicine and Physiology, University of Toronto, Toronto, ON, Canada

Cystic fibrosis is a common autosomal recessive disease caused by variants in the cystic fibrosis transmembrane conductance regulator (*CFTR*) gene, which encodes for an ion channel, CFTR, that is involved in regulation of the water–electrolyte balance on the surface of many organ systems, including the upper and lower airways, intestine, pancreas, biliary tree, cervix, vas deferens, and sweat glands (1). Thickened pulmonary secretions and reduced mucociliary transport causing pulmonary complications are the most debilitating manifestations of this multisystem disease. Intestinal manifestations such as pancreatic insufficiency and fat malabsorption affect 80% of those with cystic fibrosis and are also responsible for considerable morbidity, including malnutrition, growth failure, and fat-soluble vitamin deficiencies. High-calorie, high-fat diets and pancreatic enzyme replacement are standard therapy in patients with pancreatic insufficiency. With the introduction of CFTR modulator therapy, clinical trials have demonstrated the potential to prevent or delay pancreatic insufficiency if treatment is started at an early age, before there is irreversible pancreatic damage.

The pathogenesis of malabsorption and malnutrition in cystic fibrosis is multifactorial, with a major contributing factor being exocrine pancreatic insufficiency but also due to several other abnormalities in the intestinal lumen and factors affecting intestinal motility (2). These include abnormal functioning of Brunner’s glands, acidification of the intestinal lumen impairing pancreatic enzyme function, disrupted micelle formation and absorption secondary to bile abnormalities aggravated by cholestasis, abnormally thick mucous in the intestines affecting absorption and causing stasis and possibly a biofilm. It is fair to say that fat malabsorption has not been attributed directly to CFTR protein deficiency per se. Persistent cystic fibrosis fat malabsorption has been well described (3).

Teng *et al.* studied fat absorption, post lipid challenge lymph and plasma triglyceride (TG) excursion, chylomicron secretion and their clearance from the circulation and chylomicron size in mice harboring the CF(G542X) mutation, in an animal model of human cystic fibrosis. In addition, they examined FFA trafficking across small intestinal epithelium in primary small intestinal organoids from cystic fibrosis (CF) mice. The authors demonstrated the following in their CF mouse model versus appropriate controls. A. No evidence of defective pancreatic lipase hydrolysis of luminal lipids or fat malabsorption. B. Significant defect in dietary TG absorption from intestine into blood. C. Reduced appearance rate of labeled dietary TG and apoB (particle numbers) in the blood. D. Small-sized, denser chylomicrons that are cleared more rapidly from the circulation. E. Defective FFA trafficking across small intestinal epithelium.

These findings led them to conclude that pancreatic insufficiency leading to defects in luminal lipase or emulsification is not the major defect leading to CF nutritional deficiencies. Instead, they concluded that the defect lies in FA absorption, followed by defective chylomicron formation and metabolism.

These are carefully performed studies, using an animal model relevant for human CF, and the authors have examined several very relevant aspects of intestinal FFA transport, fat absorption, and chylomicron formation and metabolism. Results of these experiments point to a defect of FFA transport across the epithelium and their subsequent esterification in enterocytes. The G542X mouse model manifests the classic CF growth defects and intestinal disease but does not appear to have severe pancreatic exocrine insufficiency. Therefore, this mouse model eliminates exocrine insufficiency as a primary cause of FA uptake defect causing fat malabsorption. These features allowed the researchers to study lipid absorption in the setting of controlled pancreatic insufficiency.

The authors have demonstrated a defect in FA absorption and chylomicron assembly, with consequent poor lipidation of chylomicrons and their subsequent rapid clearance from the blood circulation. The organoid experiments point to an abnormality in the absorptive cells themselves, which are less capable of absorbing FAs and forming chylomicrons. They have not, however, identified the precise pathogenesis of the defect. There are several possibilities that could account for the phenotype of the animal model they describe and by extension human cystic fibrosis. They have excluded defects in exocrine pancreatic insufficiency, enteral fat emulsification, and luminal hydrolysis as a proximal reason for FA uptake deficiency. They have shown that uptake of FA might be a reason for the production of reduced amounts of small chylomicrons. FA malabsorption could be secondary to changes in the unstirred water layer on the enterocytes, reduced FA re-esterification, or increased intracellular oxidation, with diversion away from synthetic pathways.

Have the authors demonstrated a primary defect in chylomicron assembly and secretion? Chylomicron lipidation is clearly defective in this mouse model, with consequent secretion of smaller, lipid-poor chylomicrons that are subsequently rapidly hydrolyzed and cleared from the blood circulation. Marked fat malabsorption could theoretically result in defective chylomicron lipidation, assembly, and secretion. One cannot rule out a primary defect of chylomicron biosynthesis and secretion without further study but results do suggest that the primary abnormality is that of markedly reduced FA absorption by enterocytes. Interestingly, inherited disorders of metabolism, in which there is loss of individual genes involved in FA transport, do not result in appreciable defects in chylomicron metabolism (4), likely because there is redundancy of these factors and also because a large proportion of FA transport from lumen to enterocyte occurs by diffusion along a concentration gradient. This would suggest that the defect in cystic fibrosis is less likely to be due to loss of function of a crucial FA transporter and more likely to be due to the other factors mentioned above, which could be relevant even in the absence of overt pancreatic exocrine deficiency. The defect could be related to difference in the glycocalyx and transport of FA across the brush border. It is possible that the unstirred water layer is disturbed; therefore, bile acid FA micelles do not reach the enterocyte surface.

One potential clinical implication of these findings is that a high-fat diet that is widely recommended in CF may aggravate steatorrhea by overloading the absorptive capacity of the small intestine. The important findings of the Teng paper published in this issue of *Journal Lipid Research* provide new insights into the highly prevalent small intestinal manifestations of cystic fibrosis and raise further questions regarding the molecular mechanisms of these defects.

## Conflict of interest

The author declares that he has no conflicts of interest with the contents of this article.
